# EFFECTIVENESS OF HERBAL AND CONVENTIONAL MANAGEMENT INTERVENTIONS OF HEPATITIS B VIRUS

**DOI:** 10.21010/Ajidv19i.1

**Published:** 2024-10-25

**Authors:** ADEBAYO Julius John, IBRAHEEM Lawal Odula, OMORUYI Beauty Etinosa, OKUDOH Vincent Ifeanyi

**Affiliations:** 1 Department of Economics, Lead City University, Ibadan, Nigeria; 2 Bio-medicinal Research Centre, Forestry Research Institute of Ibadan, Nigeria; 3 Applied Microbial and Health Biotechnology Institute, Cape Peninsula University of Technology, Bellville, South Africa; 4 Department of Biotechnology and Consumer Science, Cape Peninsula University of Technology, Cape Town, South Africa

**Keywords:** Hepatitis B, cost-effectiveness, conventional management, phytomedicine, quality-adjusted life-years (QALYs)

## Abstract

**Background:**

Liver inflammation caused by the Hepatitis B virus (HBV) could become chronic and unresolved if untreated. Prevention and management of the disease are through vaccination and other conventional interventions. This study evaluated the cost-effectiveness of herbal management (HM) and conventional pharmaceutical management (PM) interventions in treating HBV.

**Materials and Methods:**

A purposive sampling technique was used to administer questionnaires at the University College Hospital (UCH) and Total Healthcare Diagnostic Centre (THDC). Data collection was according to a EuroQol (EQ -5D), while descriptive and inferential analyses were performed.

**Results:**

Findings revealed a high prevalence of HBV among females from ages 26 to 50. The majority discovered their health status through free HBV tests. Other than cost-effective interventions, about 73% of cases adopted HM to manage the disease. A significant difference (P<0.00) was observed in the correlation analysis of the current health status of respondents and the intervention management adopted. correlation analysis of the current health status of respondents was significantly different (P<0.01) against the intervention management adopted (P<0.00).

**Conclusion:**

The minister of health should emphasize HBV regular screening, subsidized viral load test and free vaccination in both public and private healthcare centres.

## Introduction

Hepatitis B infection caused by the Hepatitis B Virus (HBV) has become a deadly life-threatening liver infection causing both acute and chronic disease globally (Terrault *et al.*, 2018). The fast-spreading of HBV among individuals is a significant concern to medical practitioners and policymakers worldwide. In 2019, the World Health Organization (WHO) estimated about 296 million people living with chronic HBV infection, with an additional 1.5 million new cases yearly (Sonderup and Spearman, 2022). Furthermore, hepatitis B infections accounted for over 820000 deaths in 2019, most of which were attributed to cirrhosis and hepatocellular carcinoma (primary liver cancer) (Sagnelli *et al.*, 2020). The highest burden of HBV infection is concentrated in the Western Pacific Region and Africa, where 116 million and 81 million people are chronically infected. In the Eastern Mediterranean Region, approximately 16 million people are infected, 18 million in the South-East Asia Region, 14 million in the European Region and 5 million in the Region of the Americas (Terrault *et al.*, 2018). Following the United Nations (UN) report update on HBV in 2021, an estimated 9.5% prevalence, approximately 20.083 million Nigerians, are currently living with HBV disease (Ajuwon *et al.*, 2021). Previously, the reported HBV infection in Nigeria between 2000 - 2013 was estimated to be 13.6%, indicating that approximately 14% of Nigerians were carriers of HBV within that period (Musa *et al.*, 2015). According to Tessachew *et al*. (2022), the frequency of co-infection between HBV and HIV-infected patients in Ethiopia was about 46.7%. Due to the same mode of transmission of HBV and HIV, global co-infection of the viruses is 7.4% (Freeland *et al.*, 2021). Co-infection of about 1% of persons living with HBV infection (2.7 million people) are also infected with HIV Tessachew *et al.*, 2022). Globally, the prevalence of HBV infection in HIV-infected persons is approximately 7.4%. Regardless of the stage of the disease, WHO has recommended treatment for everyone diagnosed with HBV-HIV infection.

Like HIV, though more contagious, HBV is 100 times more concentrated in the blood, semen, vaginal secretions, unsafe injections or exposed sharp objects (Schillie *et al.*, 2018). Though infection is vaccine-preventable if immunized before being contracted, there is a high probability of infection right from birth, i.e., spread from mother to child during birth (Abebaw *et al.*, 2017). HBV infection acquired in infancy, and early childhood has led to chronic hepatitis in about 95% of cases, leading to WHO prioritizing infant and childhood vaccination (Mohammed *et al.*, 2022). A newly infected individual with HBV may or may not develop any symptoms. However, some people have acute achy muscles or joints, stomach pain, loss of appetite, mild fever, diarrhoea, lack of energy, constipation, dark urine, extreme fatigue, and yellowish skin or eyes (jaundice). Most people with long-term complications of acute HBV can develop advanced liver failure, leading to death (Khalid *et al.*, 2022).

Over the years, there have been controversies regarding the cure for HBV, especially between conventional and herbal medicine, which have generated public interest. Without the HBV-preventable vaccine, the spread would have been disastrous. With traditional HBV management, results have shown that the treatments depend on how long the patients have been infected (Hou *et al.*, 2020). Furthermore, the duration of the infection is categorized as short-term (acute) hepatitis B, which requires treatment to relieve the symptoms, whereas long-term (chronic) hepatitis B is often treated with orthodox medication to keep the virus under control (Sano *et al.*, 2020). This observation affirmed the findings of Hye *et al*. (2020), who reported no complete cure for HBV (Gaetan *et al.*, 2021; Hong *et al.*, 2021). However, people can live longer if appropriately managed by maintaining a healthy diet and avoiding alcoholic beverages and tobacco products. In agreement, herbal researchers concluded that herbs contain vital nutrients to treat/manage HBV infection (Awomukwu *et al.*, 2014; Egamberdieva *et al.*, 2021). In 2020, Nimat and Adejumo investigated the knowledge, attitude and practice of HBV treatment using herbal plants in South-West Nigeria. The result showed a high efficacy of about 70% complete treatment among the patients who adopted the treatment intervention.

Furthermore, another study was initiated to examine the usage of herbal medicine against viruses, including hepatitis B (Chang and Huang, 2017). They reported the inhibitory activity of the phytocompounds against the viruses to be very potent. The present study examined the cost of Phytomedicine and classical management of the hepatitis B virus among the populace in Ibadan, Nigeria.

## Materials and Methods

### Study design

The study was conducted between December 2019 and February 2021 at the University College Hospital (UCH) and Total Healthcare Diagnostic Centre (THDC) in Ibadan Southwest region of Nigeria. Ibadan is the largest city with over 6 million people within its geographical area between 7.3775^o^N and 3.9470^o^C. UCH and THDC are the busiest healthcare centres functioning for HBV viral load tests in Ibadan, and hence our data were collected from both centres. A descriptive, exploratory survey was used to determine the various management interventions of HBV, including Phytomedicine (herbal) intervention, Conventional medicine, and a combination for Phytomedicine and Conventional medicine. Data was collected using an adapted version of the EuroQol Valuation (EQ-5D) questionnaire. The EQ-5D measuring tool was developed to describe the health status of the respondents before and after using the HBV management intervention compared to the health outcomes. A few modifications were made to the EQ-5D to incorporate the respondents’ background information and socioeconomic status. In total, 100 questionnaires were distributed between UCH and THDC, resulting in 50 questionnaires per data collection point.

### Sampling population

A purposive sampling technique was employed to distribute the questionnaires among the respondents; the primary consideration for this sampling strategy was based on the researcher’s opinion and judgment regarding who could best provide the required information and who was willing to share their experiences with the researcher. Due to COVID restrictions, the study sample constituted 72 HBV patients, which was believed to be sufficient to provide the essential information needed to achieve the objective of this study. The questionnaires were retrieved from the two points (UCH and THDC) for analysis in February 2021. The inclusion criteria in this study involves voluntary adult men and women aged <15 years living and staying in Ibadan and have gone for HBV viral load test, including various treatment interventions used. Adult men and women not within the age category: unwilling to consent to participate were excluded.

### Theoretical framework

This study adopted Samuelson’s “Revealed Preference Theory (Samuelson, 1938). The theory assumes that consumers are rational in their behaviours and will always prefer the intervention that offers them more utility. It also proposed that consumers behave consistently; if they choose intervention A in a situation in which intervention B is also available, they will not select intervention B in any other case in which A is also available. Symbolically, If A > B, then B < A. It also states that if A > B and B > C in any situation, then A > C.

## Data analysis

Data were analyzed using Microsoft Excel, SPSS version 2021, to generate frequency tables and charts. Descriptive and inferential analysis were carried out using ANOVA, correlation and *R^2^*. The complex Number Model was adopted to calculate the quality-adjusted life-years (QALYs). A probability value at P < 0.05 was considered as significant level.

### Model specification

The model adopted for determining QALY in this study is the Complex Number Model (Prieto and Sacristan, 2023). This model is deeply rooted in the Pythagorean Theorem, which is of fundamental importance in Euclidean Geometry. It serves as a basis for defining a distance between 2 points. Under this condition, the QALY can only be measured by determining the modulus of these complex compositions, which are ‘Length of Life’ and ‘Utility’.

The complex number Z, ordered by a pair of real numbers (a, b) with a representation point P in a plane of coordinates (a, b) defined by ‘a’ called the real part of Z, where ‘b’ is called the imaginary part of Z, represented as shown in equation 1.


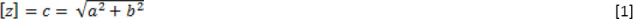


The above showed that the idea of a QALY is not different from a complex number as it is made up of a real part (length of life) and an imaginary part (Utility); this implied that utilities are intangible and not susceptible to direct observations. Thus, the QALY can be calculated as shown in Equation 2 below.


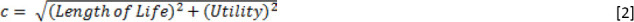


QALYs is thus obtained from the spatial relationship between the numerical values assigned to the elements in the Cartesian plane; the ratio of B/A, in this case, is equal to Equation 3 below.


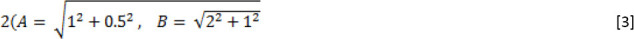


It could be inferred that A and B have the same value ratio B/A which aligned with the previous findings regarding problems and solutions in calculating QALYs.

Thus, Euclidean Geometry showing QALYs achieved using different interventions in the period becomes Equation 4 below.


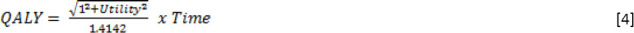


### Ethical considerations

Ethical clearance was obtained from the Biomedical Research Centre (BRC), Forestry Research Institute of Nigeria, Ibadan, Oyo State (CFGO711FRIN09). Confidentiality of individuals was maintained throughout the study.

## Results

The gender distribution of respondents consisted of 33 (45.8%) males and 39 females (54.1%), which showed that there were more female patients than male counterparts (Table 1).

**Table 1:** Sociodemographic characteristics of respondents.

Sociodemographic	Total number of respondents	
UCH	28	6
THDC	44	8

Female	39	4.1
Male	33	5.8

Age group (<15 years)	6 (2 males, 4 females)	0.3
Age group (16–20 years)	2 (males only)	0.8
Age group (21–25 years)	5 (4 males, 1 female	0.9
Age group (26–30 years)	11 (3 males, 8 females)	5.3
Age group (31–35 years)	10 (2 males, 8 females)	3.8
Age group (36–40 years)	8 (3 males, 5 females)	1.1
Age group (41–45 years)	11 (7 males, 4 females)	5.3
Age group (46–50 years)	11 (6 males, 5 females)	5.3
Age group (51–55 years)	3 (2 males, 1 female)	0.2
Age group (56–60 years)	3 (1 male, 2 females)	0.2
Age group (>60 years)	2 (1 male, 1 females)	0.8

**Total (age group)**	**72**	**00**

*Source: Researcher’s Computations, 2021*

The different age categories among respondents reported teens less or equal to 15 years of age were 6, of which 4 were females and two males. A frequency of 2 male respondents against the age bracket 16 – 20 years was recorded (Table 1).

**Figure 1:**
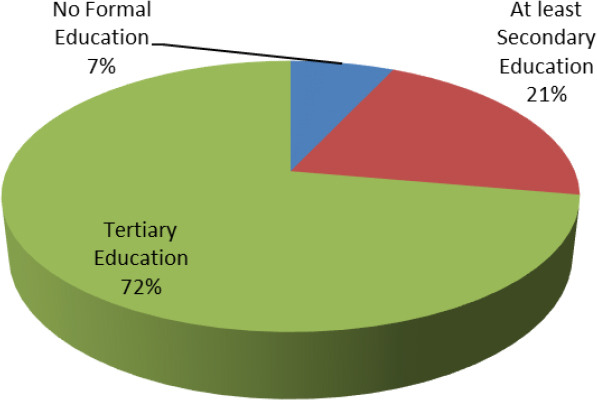
Educational status of the respondents

In addition, the level of educational attainment among the respondents revealed that 52 (72%) hold tertiary education certificates. In comparison, 15 (21%) had secondary/training certificate equivalents, and the remaining fractional number, 5 (7%) of respondents, had no formal education (Figure 1).

From Table 2, about 49% discovered their HBV status through a free test programme organized by non-governmental organizations (NGOs). Furthermore, 15% detected their HBV status through the prenatal test. In comparison, 31% were compelled to carry out a test due to frequent sickness, and 5% were already experiencing symptoms that demanded carrying out an HBV test.

**Table 2:** How respondents discovered their HBV status

Means of discovery status	No. of respondents	Percentage of respondents
Free test by NGO	35	49
Prenatal test	11	15
Test due to frequent sickness	22	31
Test due to symptom	4	5

**Total**	**72**	**100**

*Source: Researcher’s Computations, 2021*

**Figure 2:**
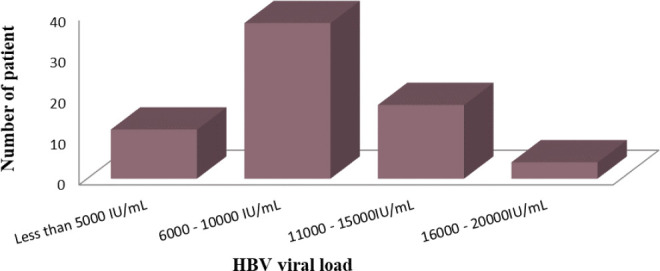
Viral load among respondents before any management intervention

The viral load among respondents was measured using international units per millilitre (IU/mL), an arbitrary amount of substance agreed upon by scientists and doctors. A millilitre is a measure of volume equal to one-thousandth of a litre (Figure 2). This survey revealed that 16.6% (12) of the respondents had their viral load below or equal to 5000 IU/mL, 52.7% (38) of respondents were between 6000 -10000 IU/mL, respondents between 11000 – 15000 IU/mL and 16000 – 20000 IU/mL constituted 25% (18) and 5.5% (4) respectively.

**Figure 3:**
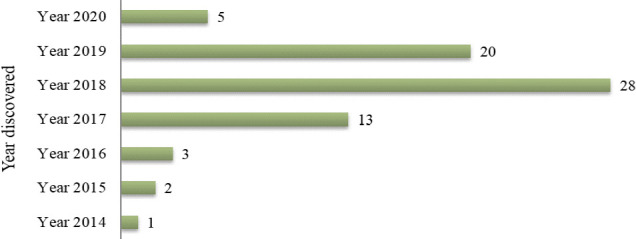
The year respondents confirmed their HBV status

The year respondents discovered their health status; three confirmed their status in 2016, followed by 28 and 20 cases in 2018 and 2019, respectively, at UCH & THDC as shown in Figure 3.

The cost of the viral load test varies among the respondents, as shown in Table 3. The test results usually take two weeks to 2 months, and there are situations where respondents must run the test many times to examine the effectiveness and Utility used during a particular management intervention. Investigation showed that 22% (16) of the respondents expended N41000/94USD – N60000/138USD on the viral load test (VLT).

**Table 3:** Respondent’s expenditure on viral load tests only

Expenditure on viral load test only	US Dollars equivalent (1 Naira=0.0023USD)	No. of respondents	Percentage of respondents
N41000 – N60000	94 – 138	16	22
N61000 – N80000	140 – 184	29	40
N81000 – N100000	186 –230	5	7
N101000 – N120000	232 –276	13	18
N121000 – N140000	278 – 322	3	4
N141000 – N160000	324 – 368	3	4
N161000 – N180000	370 – 414	1	2
> N200000	> 460	2	3

**Total**		**72**	**100**

*Source: Researcher’s Computations, 2021*

**Figure 4:**
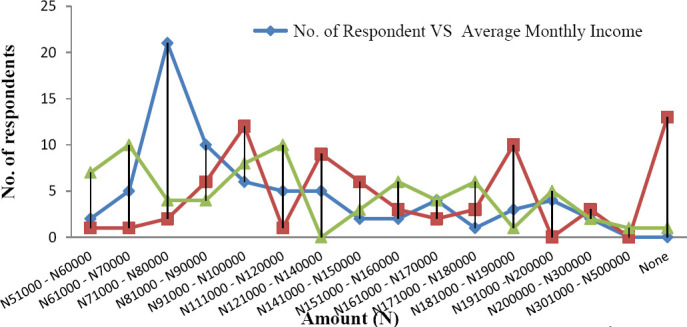
Respondent income vs average expenditure on treatment and willingness-to-pay

Different from the cost of treatment/management, and the willingness to pay, 29 (40%) yielded spending between N61000/140USD – N80000/184USD, while 13 (18%) claimed that, in total, a sum of N101000/232USD – N120000/276USD was expensed to VLT (Table 3). The average monthly income of respondents, the average expenditure on treatment/management, and willingness-to-pay concerning the intervention adopted are shown in Figure 4.

Costs incurred on treatment/management of HBV were presented according to the intervention adopted, either conventional pharmaceutical management (PM) or herbal management (HM). Two respondents expended between N51000/117USD – N60000/138USD for HM; no respondent accounted for PM and a mix of HM+PM. In the cost range of N61000/140USD – N70000/161USD, four respondents constituted HM and one for PM. None of the respondents adopted the mix of HM+PM. The statistical analysis showed that PM had an increased cost of treatment/management compared to other interventions identified in this study. However, it may not be sufficient to say that other interventions, such as HM and HM+PM mix, are affordable or cheap in the treatment/management of HBV. Still, the increased marginal Utility (MU_h_/P_h_) of HM is greater than the MU_P_/P_p_ of PM combined. The reduced cost of HM in the treatment/management of HBV over other interventions demonstrates cost-effectiveness. Respondents were more willing to go for higher-cost herbal management, HM. At the highest amount of N400000/920USD – N500000/1150USD, three respondents still adopted HM, and no one was willing to pay that amount for other interventions in this category.

**Figure 5:**
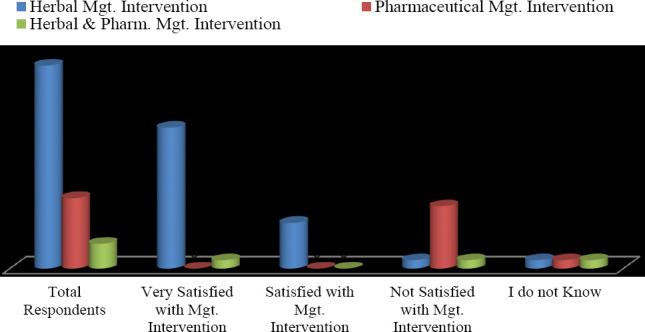
Utility derived from the HBV management intervention

All the 11 respondents who were ‘satisfied’ belonged to herbal intervention. Of 19 respondents who were ‘not satisfied’, 15 accounted for PM, while two represented HM and two recorded against the Combination of HM+PM (Figure 5). Respondents adjudged ‘neutral’ for the intervention adopted were six, which were further distributed to HM (2), conventional PM (2), and HM+PM combined (2).

**Figure 6:**
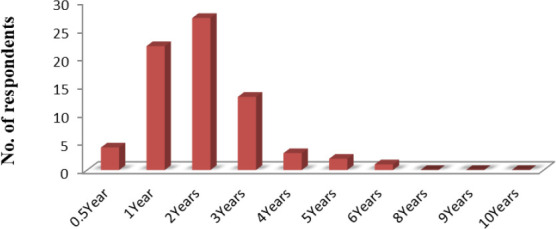
Year lived by respondents in the state of health

The lived years (LY) variable represented the number of years the respondents lived in the HBV health status through the time of this study, though dependent on the viral load (VL) level before an appropriate treatment/management intervention was adopted (Figure 6). Respondents who lived in health conditions for 0.5 years were 5.5% (4), 30.5% (22) lived for one year, while the majority (27) who lived for two years comprised 37.5%. A significant number of the respondents (18%) who lived in the health condition for three years were 13 in total; three respondents recorded having lived in the health state for four years, 4.1%. However, respondents who have lived for five years (2.7%) and six years (1.4%) accounted for two and one respondent, respectively.

The EuroQol Health Valuation (EQ-5D) tool was adopted to measure the health status of the respondents based on the treatment/management intervention adopted. The measuring tool was designed using a scale of ‘0 – 100’, where:

*0, worse health condition or death; 10 – 50, bad health condition; 60 – 70, significant improvement/ rapid recovery from HBV; 80 – 100, the respondent is in complete health status in which the viral load has shown non-reactive to HBV as at when scanned*.

This scale was also used as the decision threshold for classifying respondents based on their health status. There were three respondents in the category of 10% health status; coincidentally, these respondents adopted HM. According to data for 20% and 30% health status, there was no record concerning these groups. The respondents with 50% health status constituted 10 (13.8%), of which eight adopted PM and two adopted HM+PM. For 60% of health status, 9 (12.5%) respondents had 2 HMs, 5 PMs, and 2 HM+PM. For 70% health status, there were 12 (16.6%), ten respondents for adopted HM, 2 PMs, and 0 for HM+PM. The 80% health status comprised 29 (40.2%) respondents, with the majority (26) for HM, and 1 and 2 respondents for PM and HM+PM, respectively. The respondents with 90% – 100% health status at the study’s time were 8 (11.0%) and all constituents of HM.

The respondents (30.5%) who lived in perfect health for one year after the management intervention gained 0.8 QALY, 37.5% of respondents with two life-years (LY) gained 1QALY, while 18% of respondents in 3 life-years in perfect health gained 2.5 QALY. Respondents in 5 life-years (2.7%) and six life-years (1.4%) earned 2.5 QALY and 3 QALY, respectively (Table 4).

**Table 4:** Quality-adjusted-life-year (QALY) gained by the respondents.

No. of respondents	Percentage (%) of respondents	Life-years (LY) in health status	QALYs
4	5.5	0.5 year	0.5year gained
22	30.5	1year	0.8year gained
27	37.5	2years	1year gained
13	18	3years	2.5years gained
3	4.1	4years	2years gained
2	2.7	5years	2.5years gained
1	1.4	6years	3years gained

*Source: Researcher’s Computations, 2021*

The correlation analysis of the current health status of respondents was significantly different (P<0.01) against the intervention management adopted (P<0.00) as shown in Table 5. There was no significant difference (P<0.01) between the duration of treatment and viral load before treatment. Respondents gained quality-adjusted life-years (QALYs) as shown by the independent Utility from the intervention adopted between 1 and 3 years.

**Table 5:** Correlation analysis of the possible relationship

		Life-year in health state	Utility from intervention adopted	Treatment/Mgt intervention	Viral load before treatment	Cost of treatment
Life-year in health state	Pearson Correlation	1	0.407**	0.046**	-0.142	0.549**
	Sig. (2-tailed)		0.000	0.001	0.235	0.000
	N	72	72	72	72	72
Utility from intervention adopted	Pearson Correlation		1	0.604**	1.38	0.542**
	Sig. (2-tailed)			0.000	1.25	0.000
	N		72	72	72	72
Treatment/Mgt intervention	Pearson Correlation			1	0.046	0.632**
	Sig. (2-tailed)				0.701	0.000
	N			72		72
Viral load before treatment	Pearson Correlation				1	0.290*
	Sig. (2-tailed)					0.013
	N				72	72
Cost of treatment	Pearson Correlation					1
	Sig. (2-tailed)					
	N					72

** Significant at P<0.01;

* Significant at P<0.05; R^2^ = 72.2% *Source: Researcher’s Computations, 2021*

Setting the threshold at N80000/184USD, which was the highest frequency cost of HM, justifies its adoption, notwithstanding whether Cost/Life-year or Cost/QALY was used. The other two interventions (PM and HM+PM) would not be judged cost-effective for all the measures (Table 6).

**Table 6:** Decision thresholds based on willingness-to-pay (WTP) for life-years (LY) and quality-adjusted life-years (QALY) and the investment decision implied depending on the ratio used.

WTP Life-Year (N,000) 1 Naira=0.0023USD	WTP QALY [USD]	C/LY<WTP C/QALY<WTP	C/LY>WTP C/QALY>WTP	C/LY<WTP C/QALY>WTP	C/LY>WTP C/QALY<WTO
<N20000/46USD	<46	2	0	0	0
N35500/82USD	82	1	0	0	0
N55500/128USD	128	0	0	0	0
N65500/151USD	151	2	2	0	0
N75500/174USD	174	2	2	4	0
N85500/197USD	197	0	0	2	0
N95500/220USD	220	3	1	1	0
N115500/266USD	266	2	2	1	0
N130500/300USD	300	4	4	0	4
N150500/346USD	346	3	3	1	0
N170500/392USD	392	4	4	0	1
> N180000/414USD	414	4	4	2	1

*Source: Researcher’s Computations, 2021*

### Discussion

This study adopted two data collection points: The University College Hospital (UCH) Ibadan and the Total Health Diagnostic Centre (THDC) Ibadan. Out of the 50 questionnaires apportioned to each point, 28 (56%) were retrieved from UCH, while 44 (88%) were returned from THDC. In total, 72 questionnaires constituted 72% of the analysis. Female respondents in this study were more than the male counterparts. Our observations correlate with recent research studies of Mohammed *et al*. (2022) and Bonolo *et al*. (2023). who reported prevalence rates of HBV in 58.4% female to 41.6% male, and 73% female to 27% male in North-eastern Ethiopia and in Periurban communities in Botswana, respectively. In contrast, the findings of Ruggieri *et al*. (2018) and Moonsamy *et al*. (2022) reported higher prevalence of HBV in male than in females in a ratio of 1:4. Between the ages of 21 and 25, four males and a female accounted for five respondents in this age bracket. The age bracket between 26 – 30 years showed a frequency of 11 respondents comprised of 8 females and three males. Furthermore, there were ten respondents for ages 31 – 35 years, two males and eight females. The high prevalence of hepatitis B virus among women respondents in this study is similar to the findings of Daka *et al*. (2022) and Kassaw *et al*. (2022), who observed that hepatitis B virus was more common among women, especially sex workers between the age bracket of 20 – 29 years.

Further examination among age brackets 36 – 40 years showed that males and females constituted 3 and 5, respectively, totaling eight respondents. Between the ages of 41 – 45 years, seven males and four females accounted for 11 respondents. Aged 46 – 50 years accounted for six males and five females, which amounted to 11 respondents. Respondents between 51 – 55 years accounted for one female and two males, and 56 – 60 years were one male and two females. The analysis revealed a rising trend in the frequencies of the respondents as the age increased but diminished as the respondents’ age declined (Table 1). This result indicated that the prevalence of HBV could be higher among respondents between the ages of 26 – 50 years in the study area (Mohammed *et al.*, 2022).

The level of literacy displayed among the respondents at different educational stages showed that both educated and non-educated were aware of the implication of HBV-positive (Wong and Khalili, 2022). The highly significant number (49%) of respondents who discovered their status through a free test by the NGOs indicates how important voluntary or free services can alleviate societal problems through public-private partnerships (PPP) or other means (Mohamed *et al.*, 2020). The statistical results from the analysis affirmed that respondents who were unemployed and without a source of income were 12, while students who were still dependents accounted for 8. In the income category, 18% (13) of respondents have no source of monthly income, except 43 out of 59 respondents earn monthly income below N200,000 ($500 @ N400/$) per month. The rate of the poverty level could have driven the respondents towards free commodities and services since there was no symptomatic information before the NGOs conducted the test. Analysis of the respondent’s viral load further revealed that 22 of 38 respondents in category 6000 – 10000 IU/mL were females, while 10 out of 18 respondents in category 11000 – 15000 IU/mL were males (Mohammed *et al.*, 2022). The viral loads of these two categories were higher and considered extremely dangerous as they possessed a high chance of liver cancer if not urgently managed.

The gulping cost of VLT only in relation to the average monthly income of respondents is a catastrophic shock on the income level, considering the present inflationary pressure, unemployment rate, and respondents with no income (Ma *et al.*, 2020). Therefore, the high cost of VLT may prevent many HBV carriers from undertaking the test to confirm their viral load level (VLL) since it is required to adopt any treatment/management intervention (Ma *et al.*, 2020). The study observed that none of the respondents spent between N71000/163USD – N80000/184USD on treatment/management of HBV. All ten respondents that expended between N81000/186USD – N90000/207USD adopted HM alone, while the HM+PM mix recorded zero. Between N91000 – N100000, four respondents exist for HM and two for PM, with none for the Combination of HM+PM. The data set representing treatment/management costs between N101000/232USD – N120000/276USD showed that four respondents adopted Phytomedicine and HM, while only one used PM and none for HM+PM. As illustrated in the analysis, two respondents chose HM, and two used PM, while only one who used the HM+PM mix expended between N121000/278USD – N140000/322USD. The cost estimate between N141000/324USD – N160000/368USD recorded nil for HM, while two respondents have recorded against the PM and HM+PM Combination, respectively. The cost price between N161000/370USD – N180000/141USD for the treatment/management of HBV showed that three respondents used PM while two adopted the HM+PM mix, and none accounted for HM alone. Furthermore, the study revealed that five respondents incurred over N180000/141 USD on PM adopted, three respondents expended the same amount on HM and none for HM+PM.

The widely adopted HM in the study area could be attributed to in-depth knowledge about intervention effectiveness (Nsibirwa *et al.*, 2020). The level of education coupled with the age of the respondents also showed that respondents were highly literate and could take decisions independently (Schmit *et al.*, 2023). The outcome of respondents who lived in perfect health after the management intervention gained valuable Quality-adjusted-life-years (QALYs), indicating that the adopted intervention was influential in the management of HBV among the populace in the study area (Xu *et al.*, 2020). This observation suggested that the decision to adopt HM would translate to 73% of cases for improved and perfect health status (Xu *et al.*, 2020). Selecting a higher threshold may only reduce the Utility derived from the adoption of the intervention but will not affect the QALYs gained.

### Limitations

The increased social impact of HBV infection in several communities in Africa calls for urgent attention, prevention, diagnosis, and treatment interventions. The high cost of conventional treatment of HBV infection in underdeveloped countries, such as Nigeria, influences the adoption of herbal remedies as alternative utility (Nsibirwa *et al.*, 2020). Some of the limitations observed in the study were the fewness of patients at both healthcare centres (UCH and THDC), due to Covid-19 restrictions. The uncertain environment of the pandemic may have interrupted the in-flow of patients and behaviour at the hospital, hence a sample size of only 72 respondence were collected in the study.

Longer waiting hours of diagnosis, fear of the outcome, and service charges were also noted as major challenges that influenced the behaviour of respondents. Despite the high literacy level, a high prevalence of HBV was observed among the female respondents. Reasons why women were a significant carrier of this infectious disease in the study could be attributed to the synergistic action of male and female sex hormones and immune responses, in addition with progression of viral cells mechanism in sex or gender disparity (Belopolskaya *et al.*, 2021).

## Conclusion

The study evaluates the high cost of conventional pharmaceutical management (PM) of HBV infection over herbal management (HM) or both (PM +HM) interventions. Respondent’s willingness to adopt HM treatment over other interventions demonstrates their rational behaviours towards cost that offers them more effective Utility and duration. Most respondents lived with viral loads of between 6000 IU/mL to 15000 IU/mL, which is considered too high and detrimental to their lives. To combat the high transmission of HBV in communities, the Minister of Health in Nigeria should collaborate with the NGOs and other public-private partners to promote the willingness of residents to participate in free HBV screening programs and health examinations, vaccination, create social media awareness in relation to the mode of transmission, symptoms, and protective measures. Most importantly, more refined laboratory studies of the various herbal plants adopted by the respondence for management/treatment of HBV would be necessary to ascertain the *in vivo* and/ or clinical safety of their usage, including the quality of life derived from the adopted intervention. Or rather implement a new specific pattern of HBV single-disease contract for the health management of patients, such as the pattern adopted in Zhejiang, Chain.

## References

[1] Abebaw TA, Aderaw Z, Gebremichael B (2017). Hepatitis B virus vaccination status and associated factors among health care workers in Shashemene Zonal Town, Shashemene, Ethiopia:A cross-sectional study. BMC Research Notes.

[2] Ajuwon BI, Yujuico I, Roper K, Richardson A, Sheel M, Lidbury B (2021). Hepatitis B virus infection in Nigeria:A systematic review and meta-analysis of data published between 2010 and 2019. BMC Infectious Diseases.

[3] Awomukwu DA, Nyananyo BL, Onukwube ND (2014). Comparative phytochemical constituents and pharmacognistic importance of the vegetative organs of some *Phyllanthus* species in South-Eastern Nigeria. International Journal of Modern Botany.

[4] Belopolskaya M, Avrutin V, Kalinina O, Dmitriev A, Gusev D (2021). Chronic hepatitis B in pregnant women:Current trends and approaches. World Journal of Gastroenterology.

[5] Bonolo BP, Motswedi A, Irene G (2023). High Prevalence of hepatitis B virus infection among people with HIV in rural and Periurban communities in Botswana. Open Forum Infectious Diseases.

[6] Chang JM, Huang KL (2017). Complementary and alternative therapies in the treatment of chronic. Hepatitis B Annual.

[7] Daka D, Hailemeskel G, Fenta DA (2022). Prevalence of Hepatitis B Virus infection and associated factors among female sex workers using respondent-driven sampling in Hawassa City, Southern Ethiopia. BMC Microbiology.

[8] Egamberdieva D, Jabborova D, Babich S (2021). Antimicrobial activities of herbal plants from Uzbekistan against human pathogenic microbes. Environmental Sustainability.

[9] Freeland C, Racho R, Kamischke M (2021). Health-related quality of life for adults living with hepatitis B in the United States:A qualitative assessment. Journal of Patient-Reported Outcomes.

[10] Gaetan L, Eloi R, Verrier MN, Thomas FB (2021). Hepatitis B virus-host interactions and novel targets for viral cure. Current Opinion in Virology.

[11] Hong X, Luckenbaugh L, Perlman D (2021). Characterization and application of precore/core-related antigens in animal models of hepatitis B virus infection. Hepatology.

[12] Hou JL, Zhao W, Lee C (2020). Outcomes of long-term treatment of chronic HBV infection with entecavir or other agents from a randomized trial in 24 countries. Journal of Clinical Gastroenterology and Hepatology.

[13] Hye WL, Jae S, Sang HA (2020). Hepatitis B virus cure:Targets and future therapies. International Journal of Molecular Science.

[14] Kassaw B, Abera N, Legesse T (2022). Sero-prevalence and associated factors of hepatitis B virus among pregnant women in Hawassa city public hospitals, Southern Ethiopia:Cross-sectional study design. SAGE Open Medicine.

[15] Khalid FK, Rasheed NA, Hussein NR, Naqid IA (2022). A study of HBV infection and its risk factors in pregnant women in Zakho city, Iraq. PLoS ONE.

[16] Ma T, Lee LY, Aw MM, Lee GH (2020). Cost-effectiveness analysis of antiviral treatment for pregnant women with high viral load to prevent hepatitis B virus vertical transmission. Singapore Medical Journal.

[17] Mohamed EA, Giama NH, Shaleh HM (2020). Knowledge, attitudes, and behaviors of viral hepatitis among recent African immigrants in the United States:A community based participatory research qualitative study. Frontiers in Public Health.

[18] Mohammad ASF, AL-Mahtab M, Khan S, Yoshida O, Hiasa Y (2022). Elimination of hepatitis by 2030:Present realities and future projections. Infectious Disease and Immunity.

[19] Mohammed H, Eshetie A, Melese D (2022). Prevalence of hepatitis B virus and associated risk factors among adults'patients at Dessie referral and Kemadults'neral hospitals in Northeastern Ethiopia. Health Science Report.

[20] Moonsamy S, Suchard M, Pillay P, Prabdial-Sing N (2022). Prevalence and incidence rates of laboratory-confirmed hepatitis B infection in South Africa, 2015 to 2019. BMC Public Health.

[21] Musa BM, Bussell S, Borodo MM, Samaila AA, Femi OL (2015). Prevalence of hepatitis B virus infection in Nigeria, 2000-2013:A systematic review and meta-analysis. Nigerian Journal of Clinical Practice.

[22] Nimat BI, Adedapo A (2020). Knowledge, attitude and practice of treatment of Hepatitis-B by natural medicine practitioners in South-West Nigeria. Journal of Primary Health Care and General Practice.

[23] Nsibirwa S, Anguzu G, Kamukama S (2020). Herbal medicine use among patients with viral and non-viral Hepatitis in Uganda:Prevalence, patterns and related factors. BMC Complementary Medicine and Therapy.

[24] Prieto L, Sacristan JA (2023). Problems and solutions in calculating quality-adjusted life years (QALYs). Health Qual Life Outcomes.

[25] Ruggieri A, Gagliardi MC, Anticoli S (2018). Sex-dependent outcome of hepatitis B and C viruses'infections:Synergy of sex hormones and immune responses?. Frontiers in Immunology.

[26] Sagnelli E, Macera M, Russo A, Coppola N, Sagnelli C (2020). Epidemiological and etiological variations in hepatocellular carcinoma. Infection.

[27] Samuelson PA (1938). A note on the pure theory of consumer's behavior. Economical.

[28] Sano T, Amano K, Ide T (2020). Short-term efficacy after switching from adefovir dipivoxil and tenofovir disoproxil fumarate therapy to tenofovir alaferamide for chronic hepatitis B. Biomedical Reports.

[29] Schillie S, Vellozzi C, Reingold A (2018). Prevention of hepatitis B virus infection in the United States:Recommendations of the advisory committee on immunization practices. The Morbidity and Mortality Weekly Report (MMWR).

[30] Schmit N, Nayagam S, Lemoine M (2023). Cost-effectiveness of different monitoring strategies in a screening and treatment programme for hepatitis B in The Gambia. Journal of Global Health.

[31] Sonderup MW, Spearman CW (2022). Global disparities in Hepatitis B elimination- A focus on Africa. Viruses.

[32] Tassachew Y, Abebe T, Belyhun Y (2022). Prevalence of HIV and its co-infection with Hepatitis B/C virus among chronic liver disease patients in Ethiopia. Hepatic Medicine.

[33] Terrault NA, LOK ASF, McMahon BJ, Chang KM, Hwang JP, Jonas MM, Brown RS, Bzowej NH, Wong JB (2018). Update on prevention, diagnosis, and treatment of chronic hepatitis B:AASLD hepatitis B guidance. Hepatology.

[34] Wong RJ, Khalili M (2020). A patient-centreed hepatitis B virus (HBV) educational intervention improves HBV care among underserved safety-net populations. Journal of Clinical Gastroenterology.

[35] Xu X, Wu C, Jiang L (2022). Cost-effectiveness of hepatitis B mass screening and management in high-prevalent rural China:A model study from 2020 to 2049. Int J Health Policy Manag.

